# Anti-HLA antibody formation increases the chances of platelet refractoriness in platelet-transfused patients: a systematic review with meta-analysis

**DOI:** 10.1016/j.htct.2025.103821

**Published:** 2025-04-16

**Authors:** Luana Joana Barreto Cabral, Daniela Pereira Lopes, Eduardo dos Santos Martins Filho, Rubenilson Caldas Valois, Paula Christine Amarantes Justino Oliveira, Patrícia Jeanne de Souza Mendonça-Mattos

**Affiliations:** a Graduate Program in Multiprofessional Residency in Hemotherapy and Hematology, Pará State University (UEPA), Belém, Pará, Brazil; b Multiprofessional Residency in Hemotherapy and Hematology, Foundation Center of Hemotherapy and Hematology of Pará (HEMOPA Foundation), Belém, Pará, Brazil; c Laboratory of Immunogenetics, Foundation Center of Hemotherapy and Hematology of Pará (HEMOPA Foundation), Belém, Pará, Brazil; d Hemovigilance and Supervision Department, Foundation Center of Hemotherapy and Hematology of Pará (HEMOPA Foundation), Belém, Pará, Brazil; e Teaching Staff, Pará State University (UEPA), Belém, Pará, Brazil; f Technical Directory, Foundation Center of Hemotherapy and Hematology of Pará (HEMOPA Foundation), Belém, Pará, Brazil; g Preceptors of the State Program of Incentives to Qualification of Health Professionals – QUALIFICASAÚDE, Pará State University (UEPA), Belém, Pará, Brazil

**Keywords:** Platelet transfusion, Hla antigens, Antibody formation, Systematic Review, Meta-Analysis

## Abstract

Platelet refractoriness caused by alloimmunization to anti-HLA antibodies remains present in daily hemotherapy: the frequent need for platelet transfusions may influence the long-term survival of treated patients. This study aimed to perform a systematic review with meta-analysis to investigate the chances of anti-HLA antibody formation triggering immune-induced platelet refractoriness in platelet transfused individuals. By adopting Preferred Reporting Items for Systematic Reviews and Meta-Analyses (PRISMA) criteria, a search was conducted of publications in online databases between 1976 and July 2022. The risk of bias in the studies was assessed according to the data quality assessment proposed by the ‘A MeaSurement Tool to Assess systematic Reviews’ (AMSTAR-2) tool. Meta-analysis was performed by evaluating the Forest and Funnel Plots. From 832 published articles, 50 were read in full with 14 studies being included in this systematic review. The forest plot showed a likely low heterogeneity (I²: 12.3%; p-value = 0.32), and high odds ratio (174.57; confidence interval: 73.23–416.16) showing platelet refractoriness is triggered by anti-HLA alloantibodies. In this study, anti-HLA antibody formation contributed to an approximate 175-fold higher chance of triggering immune-induced platelet refractoriness. Some explanations about why some statistical differences were observed are offered by studies. This study demonstrates the need for developing policies to identify and monitor anti-HLA antibodies in patients, as well as for HLA matching, and makes some suggestions for future research to promote the prevention of patient sensitization due to platelet transfusions including the development of platelet refractoriness.

## Introduction

In platelet concentrate transfusions, human leukocyte antigen (HLA) compatibility between recipients and donors is not mandatory during the pretransfusion testing phase.^1^ Platelets have antigens from the ABH system, Lewis, P, I systems, Human platelet antigens (HPA), and Class I HLA antigens, but only ABO and HLA antigens and HPA are relevant for post-transfusion survival of platelets.^1^^,^^2^ Because of this, the patient may receive antigens different from his genetic heritage at each transfusion and develop sensitization to anti-HLA antibodies.^3^ This alloimmunization may trigger immune-induced platelet refractoriness, that is, an excessive consumption of platelet concentrates, without adequate therapeutic response, and with complications that can be fatal.^4^

Non-immunological causes that can trigger platelet refractoriness (60–70% of cases) include: disseminated intravascular coagulation (DIC); microangiopathic hemolytic anemia (MAHA); active bleeding; sepsis; fever; splenomegaly; graft-versus-host disease (GvHD); circulating immune-complexes; bone marrow transplantation; veno‑occlusive disease; drug-induced thrombocytopenia (antithrombotics, infectious disease agents, histamine-receptor antagonists, analgesic agents, chemotherapeutic and immunosuppressant agents, cinchona alkaloids, platelet inhibitors, antirheumatic agents, sedatives and anticonvulsant agents, and diuretic agents); platelet dose/platelet quality due to the patient's blood volume, storage temperature, improper mode of agitation and pH, and platelet age.^5^, ^6^, ^7^ However, 30–40% of cases are immune related including: HLA antibodies (80–90%), HPA antibodies (5–20%), HPA and HLA antibodies (5%), mismatched ABO antibodies, and platelet autoantibodies (e.g., platelet refractoriness related to an autoantibody to platelet glycoprotein).^5^^,^^8^

A recent prospective study of 3805 individuals pointed to pregnancy and platelet transfusion as the main risk factors for sensitization to anti-HLA antibodies: it was also observed that alloimmunization occurs mainly from platelet concentrate transfusions.^9^ Thus, anti-HLA antibodies may be present in the serum of patients with a platelet concentrate transfusion history. These alloantibodies are mostly of the IgG type^4^ and may contribute to the development of platelet refractoriness through the activation of the complement system by the classical pathway, causing the deposition of C4b and C3b and the formation of the membrane attack complex.^10^

Diagnosis can be by methods such as lymphocytotoxicity, enzyme immunoassay, platelet antigen immobilization using monoclonal antibodies, or flow cytometry. The gold standard to detect anti-HLA antibodies in blood samples is the flow cytometry technique (microspheres) called antibody reactivity panel, whose function is to identify anti-HLA antibodies, ensuring reliability, sensitivity, and specificity of the result.^4^^,^^11^^,^^12^

Thus, this review investigated the chances of anti-HLA antibody formation triggering immune-induced platelet refractoriness in platelet transfused individuals using a systematic review with meta-analysis in order to improve the statistical power of the research question.

## Materials and methods

This systematic review was conducted according to the criteria of the International Preferred Reporting Items for Systematic Reviews and Meta-Analyses (PRISMA) guidelines.^13^

### Data sources and research strategy

The PICO (population, intervention, comparison, outcome) strategy was used to formulate the following research question, "Is there evidence that, in thrombocytopenic patients, platelet transfusion with anti-HLA antibody formation increases the chances of immune-induced platelet refractoriness?" PICO was defined as: P (thrombocytopenic patients), I (platelet transfusion), C (anti-HLA antibody formation), and O (platelet refractoriness).^14^ The Web of Science (Clarivate Analytics, Philadelphia, PA, USA), Scopus, PubMed, LILACS, BVS Brasil, EBSCOhost MEDLINE, and SciELO databases were used for the search with the descriptors and keywords shown in Table 1. Publications in Portuguese, Spanish, Italian, and English were considered with articles published from 1976 to July 24, 2022 being included.

**Table 1:** Databases consulted, search strategy used with the PICO methodology in four languages, and the number of articles found.

Search strategy				Database						
	AND	AND	AND	WOS	SCOPUS	PUBMED CENTRAL	LILACS	BVS BRAZIL	Medline (EBSCOhost)	SciELO Citation Index
("aplastic anemia "OR "myelodisplasic syndromes "OR "leukemias "OR “thrombocytopenic Patients" OR “thrombocytopenia" OR "anemia aplástica "OR "síndromes mielodisplásicas "OR "leucemias "OR "pacientes trombocitopênicos "OR "trombocitopenia "OR "síndromes mielodisplásicos "OR "pacientes trombocitopénicos "O "sindromi mielodisplasiche "OR "leucemie "OR "pazienti trombocitopenici")	(platelets OR platelet OR "platelet transfusion "OR plaquetas OR "transfusão de plaquetas "OR "transfusão de plaquetas "OR "transfusión de plaquetas "OR piastrine OR "trasfusione di piastrine")	(“polytransfused platelets” OR "platelet polytransfused" OR " ineffective platelet transfusion" OR transfusion OR "plaquetas poli-transfundidas" OR "politransfusão de plaquetas" OR " transfusão de plaquetas ineficaz" OR transfusão OR "transfusión ineficaz de plaquetas" OR transfusión OR "piastrine politrasfuse" OR " trasfusione inefficace di piastrine" OR trasfusione)	(“anti-HLA” OR “HLA antibodies” OR “anti-HLA antibodies” OR “Class I HLA” OR "HLA antibody" OR "anti-HLA" OR "anticorpos HLA" OR "anticorpos anti-HLA" OR "anticorpos HLA classe I" OR "anticorpo HLA" OR "anticuerpos anti-HLA" OR "Clase I HLA" OR "anticuerpo HLA" OR "anticorpi HLA" OR "anticorpi anti-HLA")	129	5	6	4	1	686	1

### Inclusion and exclusion criteria

The selection stage included articles that met the following criteria: investigation of anti-HLA antibodies in platelet-transfused patients; cross-sectional studies or randomized clinical trials, with or without transfusion reactions to platelets, in Portuguese, Spanish, Italian, and English. The following exclusion criteria were employed: research involving neonates, and bone marrow or organ transplant recipients, case studies, meeting abstracts, reference articles, letters, editorials, notes, news, abstracts and posters from conferences, symposia, and meetings, discussions, book chapters, corrections, additions, duplications, experience reports, literature reviews, bibliographies, reprints, guidelines and retractions of publications.

### Data selection and extraction

The following data were extracted from the databases: publication year, author, publication title, abstract, keywords, journal title, institution, and country. These data were entered into the Rayyan Systems Inc. - Intelligent Systematic Review system version 0.1.0.^15^ A double-blind selection using the titles and abstracts of articles that met the inclusion criteria was made by two collaborators. A third reviewer resolved disagreements and doubts. Afterward, selected articles were read in full from the CAPES Portal Periódicos (CAFe access) and Google Scholar. Mendeley Reference Manager v. 2.76.0 was used to organize the selected articles.

### Data quality assessment

As proposed by Ma et al., the best instrument for assessing the methodological quality of a systematic review is the ‘A Measurement Tool to Assess Systematic Reviews’ (AMSTAR-2) tool.^16^ This instrument was developed and adapted from the Overview Quality Assessment Questionnaire (OQAQ), a checklist created by Sacks with the improved version was used for this paper.^17^

### Literature bias assessment

The Checklist developed by the Joanna Briggs Institute Critical Appraisal Tools “Analytical Cross-Sectional Studies” was used for cross-sectional studies and the “Checklist for Randomized Controlled Trials” was used for one single study.^16^^,^^18^ The risk of bias was considered high when positive responses were ≤49%; moderate from 50 to 69% and low risk when positive responses were ≥70%.^19^

### Data synthesis and analysis

The RStudio v.4.2.2 Build 576 interface of the R-4.2.2 for Windows program was used for the meta-analysis, using the general package for meta-analysis. The Mantel-Haenszel statistical method was used for the binary variables, and odds ratios (ORs) were obtained, given the cross-sectional nature of most of the studies. Forest plot descriptive statistics were used to compare studies, where I² <30%, 30–60%, 61–75%, and >75% are suggestive of low, moderate, substantial, and considerable heterogeneity, respectively with the significance of this heterogeneity being agreed upon at a conservative level of p-value <0.01 ^20^^,^^21^. The OR of the random or fixed (common) model was chosen. A funnel plot was also constructed to investigate the bias of all selected publications in this study.

## Results

**Figure 1:**
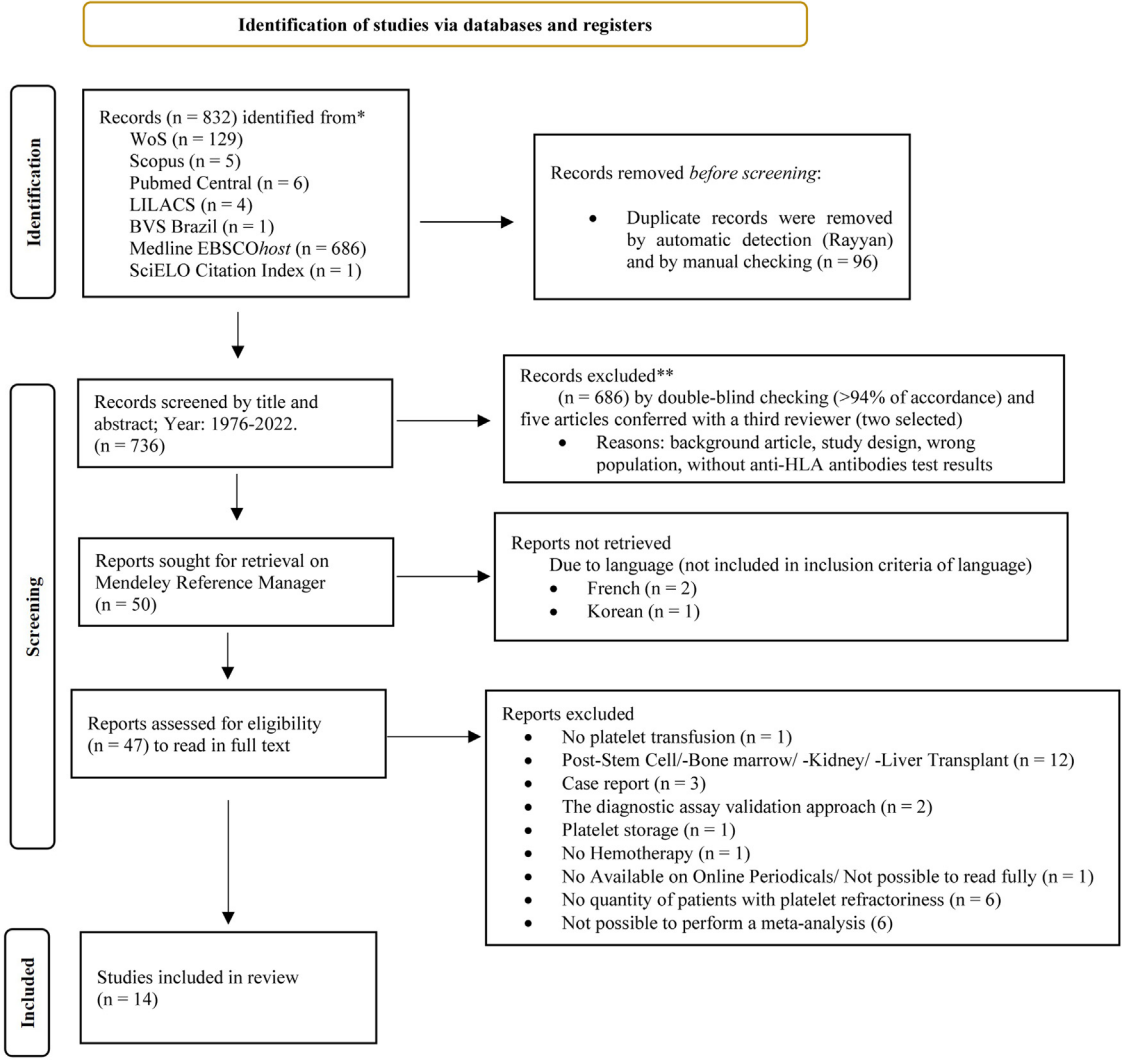
PRISMA flow diagram for the systematic review.

Table 1 shows the articles found in the different databases, the descriptors, and keywords. The PRISMA flowchart shows the entire data selection process and the number of articles included in this study (Figure 1). Of the 50 articles read in full, 33 papers were excluded for the reasons shown in Supplementary Table 1. Therefore, 14 papers (from 1976 to 2019) were included in this meta-analysis as listed in Supplementary Table 2. The risk of bias evaluated using the instruments developed by the Joanna Briggs Institute can be seen in Supplementary Tables 3 and 4. It was noted that the randomized study conducted by Hess et al. ^22^ presented a medium risk of bias with a percentage of 61.5%, while all cross-sectional studies presented a low risk of bias with percentages ranging from 87.5% to 100%.

**Figure 2:**
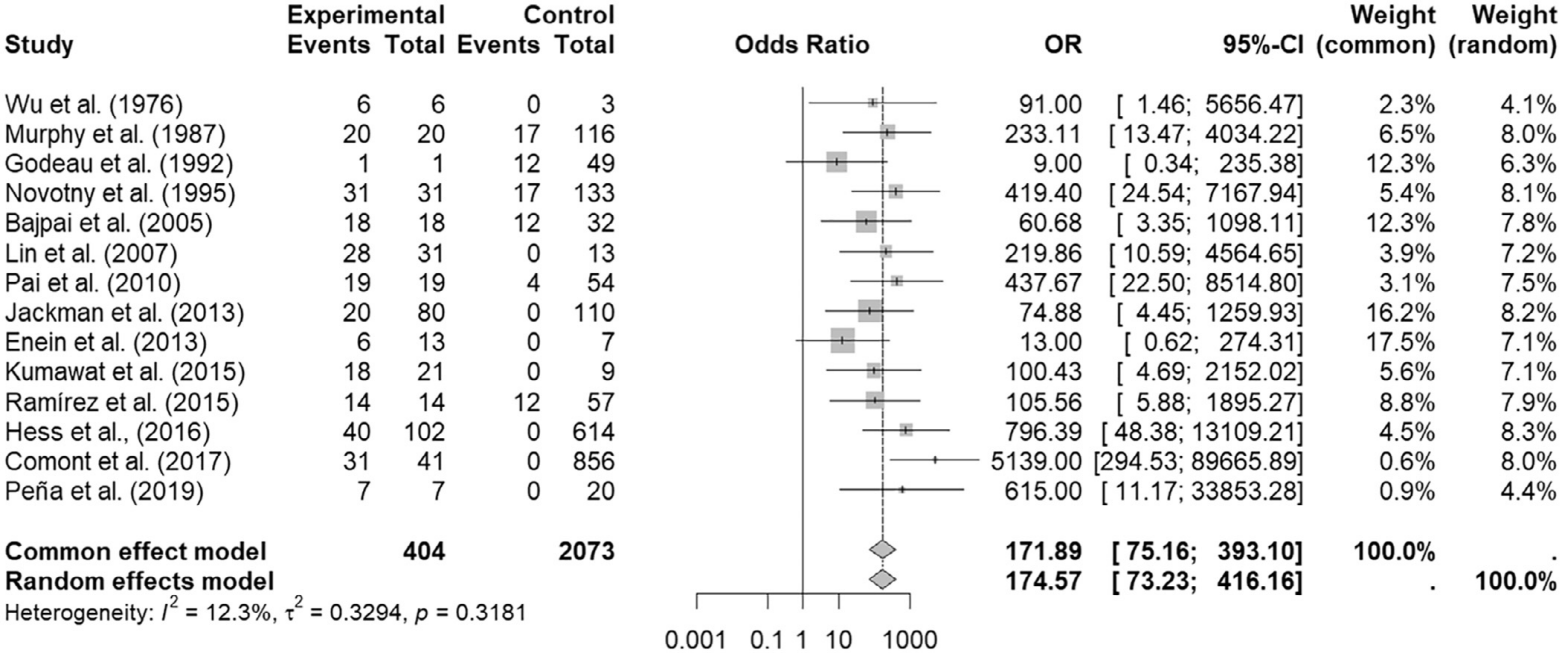
The Forest Plot of the 14 papers presenting the odds Ratio data from the quantitative data of anti-HLA antibody formation and triggering platelet refractoriness.

**Figure 3:**
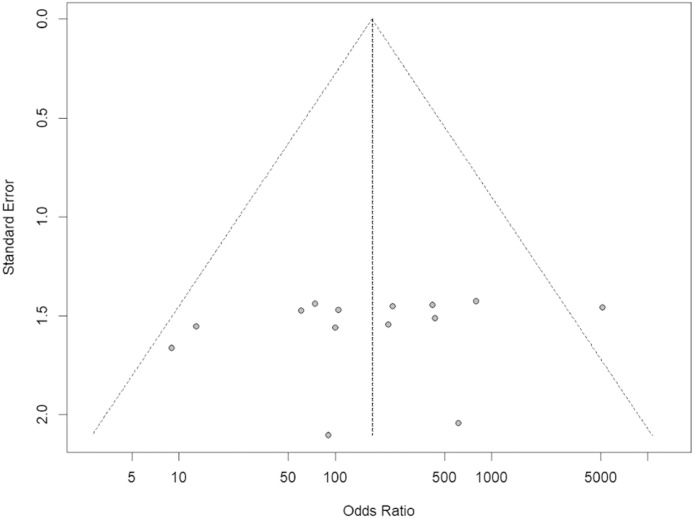
The Funnel Plot of the 14 papers presenting the risk of biased data from the quantitative data of anti-HLA antibody formation and triggering platelet refractoriness.

From the articles initially selected, due to the impossibility of performing a meta-analysis, the publications that did not present data from refractory individuals and from which it was not possible to perform the calculations were excluded (Supplementary Table 5). Table 2 shows all studies included in the meta-analysis and presents the data entered in the R program. Table 3 shows the sum of the cases for each aspect of refractoriness to anti-HLA antibodies and the performance between refractory versus alloimmunized patients using the chi-square test with Yates correction (or Fisher's exact test) in OpenEpi v. 3.01 online software.^23^ The test shows that the immunological aspect of developing refractoriness by anti-HLA antibody formation is not statistically significant compared to alloimmunized patients by non-immunological factors (p-value *=* 0.2708). Figure 2 shows the Forest Plot, which shows the low heterogeneity of the included studies (I²: 12.3%; p-value = 0.32; OR: 174.57 confidence interval [CI]: 73.23–416.16). The Funnel Plot shown in Figure 3 demonstrates the considerable symmetry in the distribution of studies (represented by dots). Therefore, it confirms the low heterogeneity and low biases of the studies. However, the study by Comont et al.^24^ was outside the pyramid, even so the standard error (1.0 <DP/√*n* < 2.0) was similar to the others. As per the funnel plot, the papers by Wu et al.^25^ (DP/√*n* > 2.0) and Peña et al.^26^ (DP/√*n* > 2.0) had the highest standard errors relative to the total nevertheless, like the others, they remained within the statistical CI.

**Table 2:** Quantitative data on the number of patients with the presence or absence of refractoriness and anti-HLA antibody formation in the 14 studies.

Author	Technique	Refractoriness^a^		Anti-HLA antibodies^a^		Total (n)	Rstudio program^b^			
		Present	Absent	Present	Absent		evtto	ntto	evcont	ncont
Wu et al.,^25^	LCY (One Lambda) and PA (Payton associates)	6	3	6	3	9	6	6	0	3
Murphy et al.,^27^	LCT (Mittal)	20	116	37	99	136	20	20	17	116
Godeau et al.,^28^	LCT (Mittal) and MAIPA (Kiefel et al.^29^	1	49	13	37	50	1	1	12	49
Novotny et al.^30^	LCT and PRA ≥ 2 0% and MAIPA (HLA w6/32)	31	133	48	116	164	31	31	17	133
Bajpai et al.^31^	LCT ≥ 2 0% (Terasaki e McClelland) and PSIFT	18	32	30	20	50	18	18	12	32
Lin et al.^32^	Flow Cytometry using donor platelet concentrates + kit FlowPRA™ (One Lambda)	31	13	28	16	44	28	31	0	13
Pai et al.^33^	Luminex Assay (LifeScreen, Tepnel Lifecodes Corporation, Stamford, CT)	19	54	23	50	73	19	19	4	54
Jackman et al.^34^	Luminex /LabScreen assay (One Lambda, Canoga Park, CA). Results with NGB. NGB > 10,8 (Class I HLA antibodies) and NGB > 6.9 (Class II HLA antibodies) were the cutoffs.	80	110	20	170	190	20	80	0	110
Enein et al.^35^	FlowPRA screening kit, (OneLambda Canoga Park, USA). And CDC	13	7	6	14	20	6	13	0	7
Kumawat et al.^36^	ELISA kit (Pakplus, GT diagnostic, USA) on three occasions (upon acceptance into the study, after 3 weeks or four transfusions, whichever occurred earlier, and at the end of 3 months)	21	9	18	12	30	18	21	0	9
Ramírez et al.^37^	Microlymphocytotoxicity for identification of HLA antibodies and Polyethylene glycol 6000 method for identification of circulating immune-complexes	14	57	26	45	71	14	14	12	57
Hess et al.^22^	CCI and PRA Class I HLA (FlowPRA Screening Kit, One Lambda Corp, Canoga Park, CA, USA);	102	614	40	776	816	40	102	0	614
Comont et al.^24^	Luminex/ LABScreen Mixed and Class I HLA Single Antigen (One Lambda). Confirmed the most reactive with LCA-CDC using a 60-cell panel.	41	856	31	866	897	31	41	0	856
Peña et al.^26^	Screening was performed with phenotyped beads from the LABScreen PRA (One Lambda Thermo Fisher; Luminex). If positive, single beads (LABScreen single antigen; One Lambda Thermo Fisher) were used. The strength of reactivity was reported as MFI. PRA was calculated using Fusion software (One Lambda Thermo Fisher). Overall, MFI≥1000 was considered positive, although standard reactivity was also considered. The percent of antibody reactivity panel calculation was determined by the online calculator optn.transplant.hrsa.gov/converge/resources/allocationcalculators.asp	7	20	7	20	27	7	7	0	20

CDC: complement-dependent cytotoxicity assay; ELISA: enzyme-linked immunosorbent assay; LCT: lymphocytotoxicity; MAIPA: monoclonal antibodies; MFI: mean fluorescence intensity; PRA: Panel Reactive Assay; NBG: normalized background.

^a^Number of refractory individuals and those who formed anti-HLA antibodies.

^b^In the RStudio program, the general package for meta-analysis package was adopted to create Forest plot and Funnel plot graphics. From the reasoning of the 2 × 2 contingency table: (I) evtto represents the number of individuals who formed anti-HLA antibodies and were refractory, (II) ntto the sum of refractory individuals who did or did not have anti-HLA antibody formation, (III) evcont the number of individuals who were not refractory but developed anti-HLA antibodies, and (IV) ncont the sum of non-refractory individuals who did or did not have anti-HLA antibody formation.

**Table 3:** Results of chi-square test with yates correction (or fisher's exact test) between refractory versus alloimmunized patients with anti-HLA antibodies.

Refractoriness^a^		Anti-HLA antibodies^a^		Total (n)	OpenEpi v. 3.01		
Present n	Absent n	Present n	Absent n		Chi-square^b^	R	NR
404	2073	333	2244	2577	**anti-HLA+**	333	2073
					**anti-HLA-**	404	2244
**Yates corrected chi-square (2-tail)**			**Fisher's exact (2-tail)**	**Odds ratio**	**Confidence interval**		
**p-value = 0.1662**			**p-value = 0.1659**	**0.8922**	**[0.7627, 1.044¹]**		

Note: ^a^From reading the complete article, the number (n) of cases with or without refractoriness and those who formed or did not form anti-HLA antibodies were noted. ^b^According to the 2 × 2 contingency table theory, the chi-square test was used considering as “disease” the refractoriness state and “exposure” anti-HLA antibody formation.

Data on the studies of Wu et al.^25^, Murphy et al.^27^, Godeau et al.^28^, Novotny et al.^30^, Bajpai et al.^31^, Lin et al.^32^, Pai et al.^33^, Jackman et al.^34^, Enein et al.^35^, Kumawat et al.^36^, Ramírez et al.^37^, Hess et al.^22^, Comont et al.^24^, Peña et al.^26^.

The Forest Plot in which the studies were included showed a probable low level of heterogeneity, represented by I² = 12.3% and p-value = 0.32. Therefore, the random or fixed model was adopted (OR: 174.57 (73.23–416.16).

The Funnel Plot showed considerable symmetry in the distribution of the studies (represented by the dots), starting from the central vertical line of the pyramid, and therefore confirms the low heterogeneity and, consequently, the low bias of the studies.

A new graphical analysis was performed, without the study by Comont et al.^24^. This showed a lesser heterogeneity (I² = 0; p-value = 0.55) and the permanence of a high OR (122.77; CI: 53.82–280.03) thereby causing the standard error of Wu et al.^25^ and Peña et al.^26^ to decrease (data not shown). However, since this new analysis confirmed the need to investigate the works of Comont et al.,^24^ Wu et al.,^25^ and Peña et al.,^26^ Figure 2, Figure 3 were considered for analysis.

Supplementary Table 2 shows all the included studies. In total, 2577 patients were investigated, and a prevalence of 12.92% (333/2577) of anti-HLA antibodies was found in refractory individuals.

Moreover, as frequent antibodies within the studied population were not one of the inclusion criteria in this study and the adopted methodologies have distinct analysis methods, only the studies by Wu et al.,^25^ Pai et al.^33^ and Peña et al.^26^ showed anti-HLA antibodies specificities (Table 4). The most common anti-HLA antibodies found in the first study were A2 and X, the last named due to the incipient serological approach. The second study presented the following highly reactive public epitopes (shared by different HLA types): 145QRT, 65QIA, 62QE, 127 K, and 163EW. Also, a common private epitope was found: 151AHA (with HLA-A11 specificities). The third study mentioned only one patient (52 years old) with two pregnancies and a regimen of platelet transfusions at: a) Day 6: HLA-B7 and HLA-B81 were the most common antibodies with high mean fluorescence intensity values; and b) Day 22: stronger mean fluorescence intensity results for HLA-B7 and HLA-B81, followed by appearances of antibodies against HLA-A68, A69, A24, A2, A23, and B67.

**Table 4:** Most common anti-HLA antibodies found in the studies.

Authors	Anti-HLA antibody
Wu et al.,^25^	A2 and X
Murphy et al.,^27^	Multispecific (it was not possible to discriminate)
Godeau et al.,^28^	Antibodies not specified, only said they were against Caucasian HLA antigens of cryopreserved lymphocytes
Novotny et al.,^30^	Antibodies not specified
Bajpai et al.,^31^	Antibodies not specified
Lin et al.,^32^	Common and rare HLA class I antigens, but not specified
Pai et al.,^33^	Highly reactive public epitopes: 145QRT, 65QIA, 62QE, 127 K, and 163EW. Most common private epitope found: 151AHA (with HLA-A11 specificities)
Jackman et al.,^34^	HLA Class I antigens, but not specified (samples from TRAP study included)
Enein et al.,^35^	HLA Class I and Class II antigens, but not specified
Kumawat et al.,^36^	HLA Class I antigens, but not specified. A significant association between HLA antibodies with (HPA)-5b/5b antibody (p-value *=* 0.033) associated with refractoriness was found
Ramírez et al.,^37^	Antibodies not specified
Hess et al.,^22^	HLA Class I antigens, but not specified
Comont et al.,^24^	HLA Class I antigens, but not specified
Peña et al.,^26^	From one patient (52 years old) with two remote pregnancies: a) Day 6 with routine PLT transfusions: HLA-B7 and HLA-B81; b) Day 22: mean fluorescence intensity stronger results of B7 and B81, followed by appearances of antibodies against A68, A69, A24, A2, A23 and B67

## Discussion

In the papers by Jackman et al.,^34^ Enein et al.,^11^ Kumawat et al.,^36^ Hess et al.,^22^ and Comont et al.,^24^ there were more participants evaluated with platelet refractoriness than with anti-HLA antibody formation. One explanation for this is by understanding that platelet alloimmunization occurs from exposure to antigens present in the platelets of the donor and absent in the patient's platelets. This alloimmunization can occur through non-immunological mechanisms such as sepsis, fever, disseminated intravascular coagulation, drugs (amphotericin-B), hypersplenism, platelet consumption by hemorrhage, or by immunological mechanisms of HLA alloantibodies, ABO alloantibodies, platelet-specific alloantibodies, as well as autoantibodies.^38^ However, in this review, the focus was on alloimmunization by the immunological mechanism of anti-HLA antibodies.

According to some papers, it would be justifiable that some patients formed anti-HLA antibodies that did not trigger platelet refractoriness. For this, one justification that exists in the literature is the description of transient anti-HLA antibody formation, which disappeared after four weeks or did not persist under remission induction chemotherapy during leukemia treatment.^27^^,^^30^

Also, in this work, the stratification of two groups was considered, prospective and retrospective cross-sectional groups, because the authors stated that they understood that, for prospective studies, the OR can overestimate the chances of the outcomes ^39^, which may cause high statistical values. A situation that was very much present in the study of Comont et al.^24^ (OR: 5139; CI: 294.53–89,665.89). Nevertheless, due to the low heterogeneity found (I² = 12.3%; p-value = 0.32) and the low number of studies that would result from stratification, the forest plot was generated with all 14 articles.

From the results of the meta-analysis, two studies deserve particular attention: Godeau et al.^28^ (OR: 9; CI: 0.34–235.38), and Enein et al.^35^ (OR: 13; CI: 0.62–274.31), because of their CIs reaching an OR <1 and Comont et al.^24^ (OR: 5139; CI: 294.53–89,665.89), because of the high OR and CI compared to the others.

The study by Comont et al.^24^ was the one that reported the most medical treatments, and perhaps, that is why it had a high OR and CI and was outside the 95% CI of the funnel plot. Moreover, its sample population was much larger than the other studies (*n* = 856). The study by Godeau et al.^28^ included patients who had not yet received platelet transfusions causing the CI to reach higher values than the other studies. In the Wu et al.,^25^ study, one can suppose that the high standard error seen in this study might be due to the old methodology adopted, low participation in the research, and the absence of transfusions received by the participants before the beginning of the investigation.

The study by Enein et al.^35^ found that the number of transfusions and the age do not influence the formation of anti-HLA antibodies that may explain the CI reaching an OR <1. In other words, the presence of anti-HLA antibodies may not have been triggered by multiple platelet transfusions. In addition, in this study, all were men, and it is well known that they are less exposed to alloimmunizations than women. The study by Peña et al.^26^ presented a possible association with a non-hemolytic transfusion reaction and performed predictive calculations of anti-HLA antibody formation. Perhaps these features increased the standard error relative to the other studies that did not take this approach. Furthermore, in some studies such as those by Jackman et al. ^34^, Enein et al.,^11^ Kumawat et al.,^36^ Hess et al.,^22^ and Comont et al.,^24^ there were more participants evaluated with platelet refractoriness than with anti-HLA antibody formation. In only two studies, Wu et al.^25^ and Peña et al.,^26^ the numbers of participants who developed the antibodies and were platelet refractory were the same.

Thus, a high OR for anti-HLA antibody formation triggering immune-induced platelet refractoriness is presented (OR: 174.57; CI: 73.23–416.16), suggesting that anti-HLA antibody formation may contribute to a 175-fold greater chance of immune-induced platelet refractoriness. The studies by Bouquegneau et al.^40^ (hazard ratio [HR]: 2.71; 95% CI: 1.98–3.72) and Kang et al.^41^ (relative risk [RR]: 2.09; 95% CI: 1.53–2.86) pointed out that anti-HLA antibodies mediated by the complement system impaired survival and increased the risk of allograft rejection. Therefore, investigating the formation of anti-HLA antibodies is very relevant because it can assist in the follow-up and therapeutic strategy of individuals after transplantation and after transfusion.^42^^,^^43^ Nevertheless, bone marrow or organ transplant recipients were not included in the selection of articles in this study because a prior allosensitization of anti-HLA antibodies could be added to sensitization by platelet donation.^44^, ^45^, ^46^

These studies do not inform which HLA antibodies were found, with only three studies by Wu et al.,^25^ Pai et al.,^33^ and Peña et al.^26^ showing specificities of anti-HLA antibodies. The studies by Wu et al.^25^ and Peña et al.^26^ showed antibodies against the HLA-A*02 allelic group. As seen in allelefrequencies.net,^47^ this is the most diverse allelic group present in all populations around the world, especially the following populations from the Americas: USA San Francisco Caucasian HLA (*n* = 220; allele frequency [AF]: 0.755), Mexico Zapotec NA-DHS_7 (G) HLA (*n* = 20; AF: 0.7), Mexico Mixe NA-DHS_6 (G) HLA (*n* = 20; AF: 0.7), Ecuador Cayapa HLA (*n* = 183; AF: 0.762), and Bolivia/Peru Quechua NA-DHS_12 (G) HLA (*n* = 21; AF: 0.785), considering frequencies over 70%. On the other hand, populations for which there were no reports of its presence were: Colombia Kogi NA-DHS_17 (G) HLA (*n* = 15; AF: not reported), Brazil Vale do Ribeira Quilombos HLA (*n* = 144; AF: 0), India West Coast Parsi HLA (*n* = 50; AF: 0), Papua New Guinea Wosera Abelam HLA (*n* = 131; AF: 0), Papua New Guinea West Schrader Ranges Haruai HLA (*n* = 55; AF: 0), Papua New Guinea Madang HLA (*n* = 65; AF: 0), Papua New Guinea Karimui Plateau Pawaia HLA (*n* = 80; AF: 0). and Papua New Guinea East New Britain Rabaul HLA (*n* = 60; AF: 0).

In the study by Pai et al.,^33^ antibodies against HLA-A*11 were detected. According to the allelefrequencies.net, this allelic group has a low distribution around the world and is only found in a few South and Southeast Asian countries (India, Myanmar, China, Vietnam, Malaysia, Thailand, Taiwan, and the Philippines). High expressions of this allele are found in Myanmar Kayar (*n* = 55; AF: 0.655). It is almost absent in the countries of sub-Saharan Africa (Natal Zulu, Burkina Faso, and Mozambique), North Africa (Morocco, Algeria, Tunisia, Sudan), and Australia.

From the point of view of clinical importance, HLA polymorphisms can generate a certain degree of susceptibility or be a protective factor against diseases since two of the most common mechanisms can affect the diversity of these alleles: 1. frequency-dependent selection, in which an individual with a rare allele may have a chance of survival in an epidemic and 2. heterozygous advantage, in which the individual may be better prepared to fight different pathogens by having a wide repertoire in the adaptive immune system, including Treg cells. Thus, HLA may play a direct role in predisposing to disease, or the polymorphism may be in linkage disequilibrium, and HLA acts as a marker.^48^^,^^49^ For example, one study appointed greater HIV vaccine efficacy for participants who expressed HLA A*02.^50^

Taking everything into account, we can see that if there is a population with similar HLA proteins and these proteins are present on platelet surfaces, allosensitization of an immunological cause is somewhat predictable and can trigger platelet refractoriness and hinder the expected transfusion response. Therefore, when all hypotheses of non-immunological causes have been ruled out in the hemotherapy service, it is essential to investigate this immunological cause or integrate it into the daily transfusion service to prevent this from happening, especially in patients who are going to undergo multiple transfusions or who have a history of transplants, pregnancy, or previous transfusions.

In eight of the fourteen studies (Table 2), the most widely used methodology for detecting anti-HLA antibodies was the cell-based complement-dependent cytotoxicity assay (CDC), a technique introduced by Terasaki and McClelland in 1964.^51^ The CDC is a technique based on cell lysis mediated by the binding of HLA molecules (expressed on the cell surface) to specific anti-HLA antibodies with subsequent activation of complement system proteins. This methodology, despite being low-cost, has some limiting factors such as different levels of expression of HLA antigens on the cell membrane, sensitivity to changes in reagents, incubation time or washing steps, and requires high cell viability and high purity, and may be susceptible to contamination with red blood cells, platelets or granulocytes.^52^^,^^53^ There are also methods for detecting antibodies using solid-phase assays such as microtiter plates like the enzyme-linked immunosorbent assay, and microbead tests in a flow analyzer, based on Luminex®/FlowPRA™ technologies. The advantages of these methods are greater sensitivity, less subjectivity in interpreting the results, and the ability to identify antibody isotypes and detect complement-fixing and non-fixing antibodies. Some disadvantages are the high cost, reagent inconsistency, particularities of the cytometer, and the conformation of HLA epitopes that can change after purification.^52^

This review did not find biases in the studies as shown by the funnel plot, revealing a good selection and database search, i.e., with minimal inclusion of gray literature. The results were shown to have low heterogeneity. Furthermore, although the investigation of immune-induced refractoriness by the formation of anti-HLA antibodies in patients has existed since the 1950s,^54^ several clinical studies are being carried out which have a high distinction in the quantitative sample population and techniques employed. Therefore, there is no methodological consensus on transfusion in the literature, and no systematic review with meta-analysis has been carried out with the current research question. In Brazil, the investigation of anti-HLA antibodies is not mandatory in pre-transfusion testing of platelet concentrates, unlike the investigation of antigens in red blood cell concentrates. This study, therefore, is concerned with individuals receiving massive platelet transfusions. Because of their underlying disease, they may be exposed to transfused platelet antigens and be sensitized with anti-HLA antibodies, potentially triggering immune-induced refractoriness with potentially life-threatening consequences.

Taking into account the reality closest to the authors (in Brazil), according to Consolidation Ordinance No 5, Annex IV (54). which deals with blood, components, and their derivatives, the mandatory pre-transfusion tests for platelet concentrates are: 1) ABO (direct and reverse) and RhD typing in the recipient's blood and 2) testing for irregular anti-erythrocyte antibodies in the recipient's blood. As can be seen, the research has an erythrocyte immunohematology approach only, but it is known that platelets have specific antigens (HPA), as well as HLA and ABO antigens. According to the same ordinance, "The pool of de-leukocytes platelet concentrates, obtained from whole blood, must contain <5.0 × 10^6^ leukocytes or each unit must contain <0.83 × 10^6^ leukocytes”.^55^^,^^56^

The double-blind, prospective, randomized, multicenter study conducted by The Trial to Reduce Alloimmunization to Platelets Study Group^57^ provided one of the first pieces of evidence on the equal effectiveness of different methods of platelet treatment by leukoreduction and ultraviolet B irradiation in preventing alloimmunization and refractoriness to platelet transfusions in patients with thrombocytopenia due to acute myeloid leukemia. This evidence has led to clinical implications and recommendations, currently applied in Brazil. This includes the use of leukoreduced and irradiated platelets in certain groups of patients as part of the transfusion protocol, intending to significantly reduce the development of antibodies and alloimmune refractoriness when bags with filters are used to reduce the incidence of antibodies present in contaminated leukocytes found in the remaining plasma, as demonstrated by Brand et al.^58^. Therefore, despite this possibility of deleukocytation, the multi-institutional study TRAP also showed that 17%–20% of patients developed anti-HLA antibodies even after leukoreduction processes ^57^.

Duquesnoy et al. pointed out that platelets with cross-reactive HLA antigen compatibility can contribute to refractiveness, since even with HLA compatibility, unsatisfactory results can sometimes occur. This can be explained by the presence of non-HLA antigens, such as HPA.^59^ Also, thrombocytopenic patients may not be able to undergo complete platelet phenotyping (including HPA) due to the insufficient number of samples. There is one caveat to these specific antigens (HPA): the genotype does not always correspond to the phenotype, especially in patients who are heterozygous for hereditary thrombopathies such as Glanzmann thrombasthenia and Bernard-Soulier syndrome. In other words, the patient may have a heterozygous genotype profile, but phenotypically have homozygosity, because one of the alleles is not expressed on the platelet surface.^60^, ^61^, ^62^

The first factor to be addressed that can contribute to the fact that this investigation of anti-HLA antibodies is not yet required as a pre-transfusion test is that it is known that HLA antigens are highly polymorphic, making it difficult to obtain several HLA-typed donors and thus provide HLA-compatible platelets for allosensitized patients. Secondly, it is important to remember that in these polymorphic HLA regions, there are epitopes shared between private and public antigens, so it is also necessary to analyze cross-reactivity, as this approach can help sustain an HLA-compatible platelet program, reducing the number of donors needed.^63^ Thirdly, there is variable expression of HLA antigens on the surface of platelets. Fourthly, the satisfactory survival of platelets due to HLA compatibility is not absolute as pointed out by the studies selected in this systematic review, due to other immunological factors like HPA or non-immunological factors that were not covered here. Fifthly, the investigation of immune-induced platelet refractoriness or the consideration of its prevention should be carried out in partnership between erythrocyte immunohematology, platelet immunohematology, and immunogenetics laboratories. At the very least, this requires alignment between institutional management, a multi-professional team, the necessary equipment for the laboratory activity, and reproducible and applicable protocols to enable collaboration, sharing, and discussion of cases between the medical and scientific communities. Unfortunately, this entire organization is not yet present in Brazil or even globally, due to inter-laboratory and intra-laboratory differences, and the distinct availability of financial resources.

It is a practice with many obstacles, but with institutional, essentially staff support, theoretical and technical training, it is possible to do a good job as seen in some hospitals like Hospital das Clínicas de Porto Alegre (HCPA) where they demonstrated a good response with the use of a platelet protocol.^64^ In this way, it will not only be possible to increase the survival of platelets in the individual but also to reduce transfusions (saving blood components that depend on the solidarity of blood donation and corroborate with the recent research on patient blood management), as well as minimizing hospitalizations in emergency rooms at transfusion agencies and reducing hospital stays when needed. Only the future will tell whether the program can be sustained through this entire support network. Those who already implanted it can be largely responsible for showing its importance and contributing to developing clinical procedures and public policies.

### Suggestions for future research

To perform a systematic review on the impact of chemotherapies in reducing alloimmunization in platelet-transfused patients. To investigate platelet allocation for transfusions on reducing alloimmunization in thrombocytopenic patients, based on the different collection bags and special procedures performed. To analyze immune and non-immune causes of platelet-refractory patients in the local reality. Adopting detection techniques of immunological causes that can be used in the economic context and available human resources to elaborate a management protocol of platelet refractoriness. After the protocol for refractory individuals is adopted, verify the effects of platelet transfusions on survival rates.

## Conclusion

This work shows that anti-HLA antibodies contribute to approximately 175-fold higher chances of triggering immune-induced platelet refractoriness. Therefore, it is interesting for hemotherapy services to investigate the existence of individuals with this condition in their local reality and with the available resources. Furthermore, this study demonstrates the need to develop public policies for identifying and monitoring anti-HLA antibodies in patients and to perform HLA matching to promote the prevention of sensitization of patients to platelet transfusions and the development of platelet refractoriness. Thus, diagnostic techniques may contribute to the excellent quality of transfusion services with the discovery and subsequent selection of more compatible platelets.

## Funding

LJBC and DPL received a scholarship from the Multiprofessional Residency Program in Hematology and Hemotherapy at the HEMOPA Foundation, through the Pará State University Multiprofessional Residency Committee (COREMU/UEPA), and a scholarship from the Pará State Government Health Professionals Qualification Incentive Program - QUALIFICASAÚDE. PJSMM and RCV also received grants from the State Program of Incentives to Qualification of Health Professionals - QUALIFICASAÚDE from the Government of Pará State, for being preceptors in the multiprofessional residency program. The content expressed in this study is of the authors and not of the Brazilian Ministry of Health, HEMOPA Foundation, UEPA, or the Government of the State of Pará.

## Conflicts of interest

The authors declare no conflict of interest.

## Appendix Supplementary materials

Image, application 1

Image, application 2

## References

[1] Dunstan R.A., Simpson M.B., Rosse W.F. Erythrocyte Antigens On Human Platelets. Absence of Rh, Duffy, Kell, Kidd, and Lutheran antigens. Transfusion (Paris). 1984;24(3):243–6. 10.1046/j.1537-2995.1984.24384225031.x 6427991

[2] Mueller-Eckhardt C., Kiełel V., Santoso S. (1990). Review and update of platelet alloantigen systems. Transfus Med Rev.

[3] Perrotta P.L., Snyder E.L., Michelson A.D. (2007). Platelets.

[4] Phelan D.L., Morris G.P., Harmening D. (2015). Técnicas Modernas em Banco de Sangue e Transfusão.

[5] Hagino T., Sato T., Tsuno N.H., Tasaki T. (2021).

[6] Novotny V.M.J. (1999). Prevention and management of platelet transfusion refractoriness. Vox Sang.

[7] Bs Rajadhyaksha, Dp Desai (2019). Navkudkar Aa. Platelet refractoriness. Global J Transfus Med.

[8] Youk H.J., Hwang S.H., Oh H.B., Ko D.H. (2022).

[9] Ma N., Guo J.P., Zhao X.Y., Xu L.P., Zhang X.H., Wang Y. (2022). Prevalence and risk factors of antibodies to HLA according to different cut-off values of mean fluorescence intensity in haploidentical allograft candidates: a prospective study of 3805 subjects. HLA..

[10] Rijkers M., Schmidt D., Lu N., Kramer C.S.M., Heidt S., Mulder A. (2019). Anti-HLA antibodies with complementary and synergistic interaction geometries promote classical complement activation on platelets. Haematologica.

[11] Enein A.A.A., El Desoukey NA, Hussein E.A.W., Hamdi M., Jamjom N.A (2013). HLA alloimmunization in Egyptian aplastic anemia patients receiving exclusively leukoreduced blood components. Transfus Apheres Sci.

[12] Kuda E., Al-Wahadneh A. (2001). Comparison of flow panel reactive assay (PRA) TM specific test with complement dependent cytotoxicity (CDC) to define the HLA antibodies specificity: a preliminary study. Saudi J Kidney Dis Transplant [Internet].

[13] Page M.J., McKenzie J.E., Bossuyt P.M., Boutron I., Hoffmann T.C., Mulrow C.D. (2021). The PRISMA 2020 statement: an updated guideline for reporting systematic reviews. The BMJ.

[14] Mamédio da Costa Santos C., Andrucioli De Mattos Pimenta C., Cuce Nobre M.R. (2007). A estratégia PICO para a construção da pergunta de pesquisa e busca de evidências. Rev Lat Am Enfermagem [Internet].

[15] Ouzzani M., Hammady H., Fedorowicz Z., Elmagarmid A. (2016). Rayyan-a web and mobile app for systematic reviews. Syst Rev.

[16] Ma L.L., Wang Y.Y., Yang Z.H., Huang D., Weng H., Zeng X.T. (2020). Methodological quality (risk of bias) assessment tools for primary and secondary medical studies: what are they and which is better?. Mil Med Res.

[17] Shea B.J., Reeves B.C., Wells G., Thuku M., Hamel C., Moran J. (2017). AMSTAR 2: a critical appraisal tool for systematic reviews that include randomised or non-randomised studies of healthcare interventions, or both. BMJ (Online).

[18] Joanna Briggs Institute (2022).

[19] Franco A., Vidigal M.T.C., de Oliveira MN, JS Nascimento CT de, da Silva RF, Paranhos L.R (2020). Evidence-based mapping of third molar techniques for age estimation applied to Brazilian adolescents – a systematic review. Res, Soc Develop.

[20] Singh S. (2017). How to conduct and interpret systematic reviews and meta-analyses. Clin Transl Gastroenterol.

[21] Pereira M.G., Galvão T.F. (2014). Heterogeneidade e viés de publicação em revisões sistemáticas. Epidemiologia e Serviços de Saúde.

[22] Hess J.R., Trachtenberg F.L., Assmann S.F., Triulzi D.J., Kaufman R.M., Strauss R.G. (2016). Clinical and laboratory correlates of platelet alloimmunization and refractoriness in the PLADO trial. Vox Sang..

[23] Dean A., Sullivan K., Soe M. (2013). http://www.OpenEpi.com.

[24] Comont T., Tavitian S., Bardiaux L., Fort M., Debiol B., Morère D. (2017). Platelet transfusion refractoriness in patients with acute myeloid leukemia treated by intensive chemotherapy. Leuk Res.

[25] Wu K.K., Thompson J.S., Koepke J.A., Hoak J.C., Flink R. (1976). Heterogeneity of antibody response to human platelet transfusion. J Clin Investig.

[26] Peña J.R.A., Makar R.S. (2019). Routine solid phase multiplex anti-HLA antibody tests predict platelet refractoriness. Am J Clin Pathol.

[27] Murphy M.F., Metcalfe P., Lister T.A., Waters A.H. (1987). Disappearance of HLA and platelet-specific antibodies in acute leukaemia patients alloimmunized by multiple transfusions. Br J Haematol.

[28] Godeau B., Fromont P., Seror T., Duedari N., Bierling P. (1992). Platelet alloimmunization after multiple transfusions: a prospective study of 50 patients. Br J Haematol.

[29] Kiefel M., Santoso V., Weisheit S., Mueller-Eckhardt C. (1987). Monoclonal antibody-specific immobilization of platelet antigens (MAIPA): a new tool for the identification of platelet-reactive antibodies. Blood.

[30] Novotny V.M.J., Van Doorn R., Witvliet M.D., Claas F.H.J., Brand A. (1995). Occurrence of allogeneic HLA and Non-HLA antibodies after transfusion of prestorage filtered platelets and red blood cells: a prospective study. Blood..

[31] Bajpai M., Kaura B., Marwaha N., Kumari S., Sharma R.R., Agnihotri S.K. (2005). Platelet alloimmunization in multitransfused patients with haemato-oncological disorders. Natl Med J India.

[32] Lin J.S., Lyou J.Y., Chen Y.J., Chen P.S., Liu H.M., Ho C.H. (2007). Unappreciated HLA antibodies in adult immune thrombocytopenic purpura. J Formosan Med Assoc.

[33] Pai S.C., Lo S.C., Lin Tsai S.J., Chang J.S., Lin D.T., Lin K.S. (2010). Epitope-based matching for HLA-alloimmunized platelet refractoriness in patients with hematologic diseases. Transfusion (Paris).

[34] Jackman R.P., Deng X., Bolgiano D., Lebedeva M., Heitman J.W., Busch M.P. (2013). Low-level HLA antibodies do not predict platelet transfusion failure in TRAP study participants. Blood [Internet].

[35] Enein A.A.A., El Desoukey N.A., Hussein E.A.W., Hamdi M., Jamjom N.A., Enein A.A.A. (2013). HLA alloimmunization in Egyptian aplastic anemia patients receiving exclusively leukoreduced blood components. Transfus Apheres Sci [Internet].

[36] Kumawat V., Sharma R., Malhotra P., Marwaha N. (2015). Prevalence of risk factors for platelet transfusion refractoriness in multitransfused hemato-oncological patients at tertiary care center in North India. Asian J Transfus Sci.

[37] Ramírez I.H., Céspedes Sánchez B.M., Ramos M.L., Campaña N.G., Sánchez M.I (2015). Refractariedad a las transfusiones de plaquetas en pacientes con enfermedades oncológicas. Correo Científico Médico de Holguín.

[38] Zago MA, Falcão RP, Pasquini R. Tratado De Hematologia. Spector N, Covas DT, Rego EM, editors. São Paulo: Atheneu; 2013. 899 p

[39] Alavi M., Hunt G.E., Visentin D.C., Watson R., Thapa D.K., Cleary M. (2020). Using risk and odds ratios to assess effect size for meta-analysis outcome measures. J Adv Nurs.

[40] Bouquegneau A., Loheac C., Aubert O., Bouatou Y., Viglietti D., Empana J.P. (2018). Complement-activating donor-specific anti-HLA antibodies and solid organ transplant survival: a systematic review and meta-analysis. PLoS Med.

[41] Kang Z.Y., Liu C., Liu W., Li D.H. (2022). Effect of C1q-binding donor-specific anti-HLA antibodies on the clinical outcomes of patients after renal transplantation: a systematic review and meta-analysis. Transpl. Immunol..

[42] Brasil M da S. 21/10/2009. 2009 [cited 2021 Dec 9]. p. 103 Portaria no 2.600, de 21 de outubro de 2009. Available from: https://bvsms.saude.gov.br/bvs/saudelegis/gm/2009/prt2600_21_10_2009.html.

[43] Brasil M da S. (2021). https://www.in.gov.br/en/web/dou/-/portaria-n-615-de-27-de-maio-de-2021-323566540.

[44] Ixtlapale-Carmona X., Arvizu A., De-Santiago A., González-Tableros N., López M., Castelán N. (2018). Graft immunologic events in deceased donor kidney transplant recipients with preformed HLA-donor specific antibodies. Transpl Immunol.

[45] Dahl J., Refsum E., Ahlen M.T., Egeland T., Jensen T., Viken M.K. (2017). Unraveling the role of maternal anti-HLA class I antibodies in fetal and neonatal thrombocytopenia—Antibody specificity analysis using epitope data. J Reprod Immunol.

[46] Muro M., Moya-Quiles M.R., Mrowiec A. (2016). Humoral response in liver allograft transplantation: a review of the role of anti-human leukocyte antigen (HLA) antibodies. Curr Protein Pept Sci.

[47] Gonzalez-Galarza F.F., Christmas S., Middleton D., Jones A.R. (2011). Allele frequency net: a database and online repository for immune gene frequencies in worldwide populations. Nucleic Acids Res.

[48] Howell W.M. (2014). HLA and disease: guilt by association. Int J Immunogenet.

[49] Robert W. (2019). A simple guide to the interpretation of the significance of the association of a disease with a particular HLA allele. Swiss Med Wkly.

[50] Gartland A.J., Li S., McNevin J., Tomaras G.D., Gottardo R., Janes H. (2014). Analysis of HLA A*02 association with vaccine efficacy in the RV144 HIV-1 vaccine trial. J Virol.

[51] Saito P.K., Yamakawa R.H., da Silva, Pereira L.C.M., da Silva, Junior W.V., Borelli S.D. (2014). Complement-dependent cytotoxicity (CDC) to detect anti-HLA antibodies: old but Gold. J Clin Lab Anal [Internet].

[52] Hahn A.B., Geoffrey Land dipABHI A., Rosemarie Strothman H.M., Blanck C.E., Phelan D.L., Adams P.W. (2000).

[53] Terasaki P.I., McClelland J.D. (1964).

[54] Dausset J., Colin M., Colombani J. (1960). Immune Platelet Iso-Antibodies. Vox Sang.

[55] Brasil M., da S. (2017). Portaria de Consolidação n^o^5, de 28 de setembro de 2017. Diário Oficial da União.

[56] BRASIL (2016). https://bvsms.saude.gov.br/bvs/saudelegis/gm/2016/prt0158_04_02_2016.html.

[57] McFarland J., Menitove J., Kagen L., Braine H., Kickler T., Ness P. (1997). Leukocyte Reduction and Ultraviolet B Irradiation of Platelets to Prevent Alloimmunization and Refractoriness to Platelet Transfusions. N Engl J Med [Internet].

[58] Brand A., Van Leeuwen A., Eernisse J.G., Van Rood J.J. (1978). Platelet transfusion therapy. Optimal donor selection with a combination of lymphocytotoxicity and platelet fluorescence tests. Blood.

[59] Duquesnoy J., Filip D.J., Rodey G.E., Rimm A.A., Aster R.H. (1977). Successful transfusion of platelets “mismatched” for HLA antigens to alloimmunized thrombocytopenic patients. Hematology.

[60] Arinsburg S.A., Shaz B.H., Westhoff C., Cushing M.M. (2012). Determination of human platelet antigen typing by molecular methods: importance in diagnosis and early treatment of neonatal alloimmune thrombocytopenia. Am J Hematol.

[61] Kannan M., Yadav B.K., Ahmad F., Biswas A., Saxena R. (2009). Modulation of clinical phenotype of Glanzmann's thrombasthenia by thrombogenic mutations. Clinica Chimica Acta.

[62] Koskela S., Kekomäki R., Partanen J. (1998). Genetic polymorphism in human platelet glycoprotein GP Ib/IX/V complex is enriched in GP V (CD42d). Tissue Antigens.

[63] Duquesnoy R.J., White L.T., Fierst J.W., Vanek M., Banner B.F., Iwaki Y. (1990). Multiscreen serum analysis of highly sensitized renal dialysis patients for antibodies toward public and private class I HLA determinants. Implications for computer-predicted acceptable and unacceptable donor mismatches in kidney transplantation. Transplantation.

[64] Fagundes I.S., Franz J.M., Jobim M.S., Arend A., Merzoni J., Cardone J.M. (2020). Diagnosis and treatment of immunological platelet refractoriness by histocompatibility. Hum Immunol [Internet].

