# Supplementary material for: Anti-HLA antibody formation increases the chances of platelet refractoriness in platelet-transfused patients: a systematic review with meta-analysis
Source: Hematol Transfus Cell Ther. 2025 Apr 16;47(2):103821. doi: 10.1016/j.htct.2025.103821 (PMC12032179; doi:10.1016/j.htct.2025.103821)
Supplement: Supplementary file 1 [file mmc1.docx]

**Supplementary Table 1 –** Information from the 33 papers excluded from this systematic review after being read entirely, organized according to the reason for exclusion.

| **Year** | **Locality** | **Journal** | **Title** | **Authors** | **Reason for exclusion** |
| --- | --- | --- | --- | --- | --- |
| 2015 | China, Zhejiang | Platelets | Successful use of rituximab in platelet transfusion refractoriness in a  multi-transfused patient with myelodysplastic syndrome | Yu *et al*. | Case Report |
| 2016 | EUA, Lousiana | Journal of the Lousiana State Medical Society | Absolute Platelet Refractoriness associated with HLA antibodies: A Case Report | Vora *et al*. | Case Report |
| 2019 | EUA, NY | Am J Clin Pathol | HLA-Mediated Platelet Refractoriness | Schmidt *et al*. | Case Report |
| 1985 | EUA, Vermont | Transfusion | Platelet adherence and phagocytosis  A method for the detection of platelet antibodies | B. R. Macphersoann D N. Cornish | The diagnostic assay validation approach |
| 1987 | EUA, CA | Blood | A Specific Assay for Anti-HLA Antibodies:  Application to Platelet Donor Selection | Millard *et al*. | The diagnostic assay validation approach |
| 1988 | EUA, Boston | Blood | Stability of Antigens on Leukocytes in Banked Platelet Concentrates: Decline in HLA-DR Antigen  Expression and Mixed Lymphocyte Culture Stimulating Capacity Following Storage | Mark E. Sherman and Walter H. Dzik | Platelet storage |
| 1994 | Australia | Pathology | Cross-matched platelets in boné marrow transplantation | Rule *et al*. | Post-Stem Cell Transplant |
| 1997 | Chile | Revista Méd Chile | Aloinmunización plaquetária em pacientes onco-hematológicos politransfundidos: estudio prospectivo em adultos y niños | Pereira *et al*. | No Available on Online Periodicals/ Not possible to read fully/Couldn’t reach the author by e-mail |
| 2014 | Turkey, Istanbul | International Journal of Rheumatic Diseases | Increased frequency of class I and II anti-human leukocyte  antigen antibodies in systemic lupus erythematosus and  scleroderma and associated factors: a comparative study | Tozkir *et al*. | No Hemotherapy |
| 2015 | EUA, Atlanta | British Journal of Haematology | Red blood cell transfusions are associated with HLA class I but  not H-Y alloantibodies in children with sickle cell disease | Nickel *et al*. | No platelet transfusion |
| 1981 | EUA, CA | Blood | Suppression of Transfusion-Related Aloimmunization in Intensively Treated Cancer Patients | Thomas V. Holohan, Paul I. Terasaki and Albert B. Deisseroth | Post- Bone Marrow Transplantation |
| 1993 | Finland | Vox Sanguinis | Systematic Use of Leukocyte-Free Blood Components to Prevent  Alloimmunization and Platelet Refractoriness in Multitransfused Children with Cancer | U. M. Saarinen | Post-Stem Cell Transplant |
| 1996 | Spain | British Journal of Haematology | Clinical and laboratory factors associated with platelet  transfusion refractoriness: a case-control study | Iñaki Alcorta, Arturo Pereira and Antonio Ordinas | Post-Stem Cell Transplant |
| 2013 | Australia | British Journal of Haematology | The mean fluorescence intensities of anti-HLA antibodies  detected using micro-bead flow cytometry predict the risk  of platelet transfusion refractoriness | Beligaswatte *et al*. | Post-Stem Cell Transplant |
| 2014 | China, Beijing | Transfusion Medicine | Prevalence of platelet-specific antibodies and efficacy  of crossmatch-compatible platelet transfusions  in refractory patients | Jia *et al*. | Post-Stem Cell Transplant |
| 2019 | Sweden | Transfusion Practice | HLA-selected platelets for platelet refractory patients with  HLA antibodies: a single-center experience | Karlström *et al*. | Post-Stem Cell Transplant |
| 2020 | France | Transfusion | Platelet transfusion refractoriness and anti-HLA immunization | Blandin *et al*. | Post-Stem Cell Transplant |
| 2020 | Australia | Transfusion | The burden of immune-mediated refractoriness to platelet  transfusions in myelodysplastic syndromes | Cheok *et al*. | Post-Stem Cell Transplant |
| 2020 | EUA, Minnesota | Transfusion | Human leukocyte antigen (HLA)-incompatible mean  fluorescence intensity-selected platelet products have  corrected count increments similar to HLA antigen matched  platelets | Karafin *et al*. | Post-Stem Cell Transplant |
| 2010 | China, Guangzhou | Transfusion Medicine | Establishment of platelet donor registry improves the treatment  of platelet transfusion refractoriness in Guangzhou region  of China | Xia and Ye *et al*. | Post-Kidney Transplant |
| 2021 | Brazil, São Paulo | Transfusion and Apheresis Science | Autoimmune pancytopenia after liver transplantation: A case report | Barros *et al*. | Post-Liver Transplant |
| 1997 | London | British Journal of Haematology | Pilot study of HLA alloimmunization after transfusion with  pre-storage leucodepleted blood products in aplastic anaemia | Killick *et al*. | No quantity of patients with platelet refractoriness |
| 2017 | China | Transfusion Medicine | Analysis of platelet-reactive alloantibodies and evaluation of  cross-match-compatible platelets for the management of  patients with transfusion refractoriness | Wang and Xia *et al*. | No quantity of patients with platelet refractoriness |
| 2018 | India | Transfusion Medicine | Antibodies to human platelet antigens form a significant  proportion of platelet antibodies detected in Indian patients  with refractoriness to platelet transfusions | Abraham *et al*. | No quantity of patients with platelet refractoriness |
| 2019 | United Kingdom | Transfusion Medicine | Impact of Human Leucocyte Antigen epitope matched platelet transfusions in alloimunised aplastic anaemia patients | Kallon *et al*. | No quantity of patients with platelet refractoriness |
| 2020 | Brazil, Porto Alegre | Human Immunology | Diagnosis and treatment of immunological platelet refractoriness by  histocompatibility | Fagundes *et al*. | No quantity of patients with platelet refractoriness |
| 2021 | Minnesota, USA | Transfusion | Prozone rates in the solid-phase platelet crossmatch assay  and correlation with class I HLA antibody levels | Juskewitch *et al*. | No quantity of patients with platelet refractoriness |
| 2001 | Germany | Transfusion | Platelet alloantibodies in transfused patients | Kiefel *et al*. | Not possible to perform a meta-analysis |
| 2007 | Japan | Transfusion Medicine | Comparison of acute non-haemolytic transfusion reactions in female and male Patients receiving female or male blood components | Imoto *et al*. | Not possible to perform a meta-analysis |
| 2008 | Brazil, Fortaleza | Revista Brasileira de Hematologia e Hemoterapia | Aloimmunity against HLA class I antigens in patients with myelodisplastic syndrome and aplastic anemia | Arruda *et al*. | Not possible to perform a meta-analysis |
| 2010 | Japan | Transfusion Medicine | Acute non-hemolytic transfusion reactions and HLA class I  antibody: advantages of solid phase assay compared with  conventional complement-dependent assay | Imoto *et al*. | Not possible to perform a meta-analysis |
| 2020 | France | Transfusion | Dominant immune response to HLA-B57/B58 molecules  after platelet transfusion | Coombs and Hassen *et al*. | Not possible to perform a meta-analysis |
| 2022 | Beijing, China | HLA | Prevalence and risk factors of antibodies to HLA according  to different cut-off values of mean fluorescence intensity  in haploidentical allograft candidates: A prospective study  of 3805 subjects | Ma and Guo *et al*. | Not possible to perform a meta-analysis |

Source: Elaborated by the authors (2022).

**Supplementary Table 2 -** Summary of the main characteristics of the 14 studies selected after an individual review of the papers included in this systematic review.

| **Year/**  **Locality** | **Journal** | **Title** | **Authors** | **Participants/**  **Period Time** | **Study Type** | **Detection Techniques of acHLA** | **Financial Support** | **Outcomes** |
| --- | --- | --- | --- | --- | --- | --- | --- | --- |
| 1976  Iowa, EUA | Journal of Clinical Investigation | Heterogeneity of antibody response to human platelet transfusion | Wu, *et al*. | 9 (7 female + 2 male) with medullary hypoplasia due to AA (2) and acute leukemia (7) without previous platelet transfusion  N.D. | Cross-sectional Prospective | LCY (One Lambda) and PA (Payton associates) | Supported in part by a research contract (NOI-HL-2957) with the National Heart and Lung Institute | The amount of platelets transfused to trigger alloimmunization is variable; one group showed anti-HLA alloantibodies with the presence of platelet aggregation and another with only platelet aggregation; allo-HLA identified as IgG; adopt another technique to identify allo-HLA; PA is suggested as a cross-matching. |
| 1987  London | British Journal of Haematology | Disappearance of HLA and platelet-specific antibodies  in acute leukaemia patients  alloimmunized by multiple transfusions | Murphy *et al*. | 154 patients with Acute Leukemia and under remission induction chemotherapy  N.D. | Cross-sectional Retrospective | LCT (Mittal, 1978) | N.D. | Only a small proportion of all immunized patients had persistently detectable HLA antibodies; the proportion of patients with the antibodies detected during transfusions was no greater than those who stopped the transfusion regimen or switched to HLA-matched platelets; 25 of 27 patients who developed HLA antibodies during induction therapy had transient alloimmunization. |
| 1992  France | British Journal of Haematology | Platelet alloimmunization after multiple transfusions:  a prospective study of 50 patients | Godeau *et al*. | 50 (24 females + 26 males) with aplastic anemia (2), ALL (8), AML (14), multiple myeloma (5) Hodgkin's disease (3), non-Hodgkin's lymphoma (15), solid tumor (3) without previous platelet transfusions  N.D. | Cross-sectional Prospective | LCT (Mittal, 1968) and MAIPA (Kiefel et al., 1987) | N.D. | Of the 50 patients, 13 detected acHLA; the MAIPA technique detected only 4/7 patients with HLA IgG antibody; the major responsible for alloimmunization was HLA rather than HPA; antibodies appeared even with intensive chemotherapy and irradiation regimen; it was not possible to observe the difference between platelet concentrate of whole blood and platelet concentrate by plateletpheresis; the combined use of LCT and PSIIFT techniques demonstrated one individual who developed non-complement-dependent acHLA; alloimmunization was undetectable during aplasia; antibodies were detected within 5-15d and 30-80d after platelet transfusion. |
| 1995  Norway | Blood | Occurrence of Allogeneic HLA and Non-HLA Antibodies After Transfusion  of Prestorage Filtered Platelets and Red Blood Cells: A Prospective Study | Novotny *et al*. | 164 (107 males and 57 females) with aplastic anemia, ALL, NHL, HD, SMD and other hematological diseases  1989 to 1992 | Cross-sectional Retrospective | LCT and PRA ≥ 20% and MAIPA (HLA w6/32) | N.D. | Of 48/164 patients with acHLA, 29 were on admission, and 19 patients during the study period. 3/112 with a negative history of transfusions or pregnancy were as follows: one male required HLA-matched transfusion after 6 weeks; the second patient developed transient anti-HLA antibodies disappearing after 4 weeks and could receive platelets from random donors; the third patient was a child who developed antibodies with PRA >50% within a week of 6 transfusions (15 units) with a history of leukoreduced packed red cells and post-storage filtered platelets; HLA with PRA >40%, demonstrated in 31 patients, was significantly associated with platelet refractoriness, in contrast to 17 non-refractory patients with PRA <40%. |
| 2005  India | The National Medical Journal of India | Platelet alloimunization in multitransfused patients with haemato-oncological disorders | Bajpai *et al*. | 50 patients (31 male, 19 female), included: aplastic anemia (16), acute leukemias (23), CML (6), NHL (2), myelofibrosis (1)  N.D. | Cross-sectional Retrospective | LCT ≥ 20%  (Terasaki and McClelland) and PSIFT | N.D. | 47 patients were acHLA negative. Of these, 27 became positive after 3-4 weeks post-transfusion. 3 patients had acHLA+ at pre (PRA = 6%, 6%, 10%, respectively) and post (16.6%, 26.7%, 100%), suggesting pre-existing ac and emergence of new ones. 20 were acHLA-. The incidence of acHLA by LCT was 60% and PRA between 3%-100%. PSIFT was positive in 19 patients, of these 16 also by LCT and 3 on PSIFT alone. The incidence by PSIFT was 38%. There was no statistical significance between women with a previous pregnancy and high incidence of acHLA (83%) with those with no history (60.5%). Statistical significance of acHLA incidence occurred in those with a transfusion history (91.7%) over those with no history (57.9%). No correlation occurred between transfusions with acHLA formation and between the underlying disease and this formation. Seven patients did not require platelet transfusions and were not included in the statistics for response to platelet therapy. Response to therapy was significantly lower in patients with acHLA+ (p<0.05). 7/11 patients with PRA<20% had a response to therapy, while 1/19 patients with PRA>20% who had a response. |
| 2007  Taiwan | Journal Formosan Medical Association | Unappreciated HLA Antibodies in Adult Immune Thrombocytopenic Purpura | Lin *et al*. | 44 ITP patients (28 women and 16 men)  N.D. | Cross-sectional Retrospective | Flow Cytometry using donor platelet concentrates + kit FlowPRA^TM^ (One Lambda) | This study was supported, in part, by grant VGH 93-A-039 from Taipei Veterans General Hospital (Taipei, Taiwan) | By pooled cytometry, 31/44 patients had reactive results while 28/44 showed reactivity by the commercial kit. Of the 28, no statistically significant correlation occurred with TX history or gender and in patients with a history of pregnancy, the appearance of Class I acHLA also did not correlate with pregnancy. Six men and four women without a history of TX and pregnancy had Class I acHLA. FlowPRA^TM^ has some advantages over lymphocytotoxicity testing such as: time reduction using commercially available beads already used for acHLA detection in renal patients to predict graft rejection, but disadvantages such as high cost and the inability to differentiate auto from alloantibodies. |
| 2010  Taiwan | Transfusion Practice | Epitope-based matching for HLA-alloimmunized platelet  refractoriness in patients with hematologic diseases | Pai *et al*. | 73 patients (33 women and 40 men) with hematological diseases (AML, CML, ALL/CLL, MDS, SAA, other). 23/73 were alloimmunized with acHLA and 9/23 had their data analyzed due to low ICC after two random platelet transfusions and followed up for 7 months  Sept/2006 to May/2007 | Cross-sectional Prospective | Luminex assay (LifeScreen, Tepnel Lifecodes Corporation,Stamford, CT) | This study was supported by Grant NSC96-2320-B-002-039-MY3 from the National Science Council, Taiwan, and by the Taiwan Blood Services Foundation Research Program, Taiwan | 23/73 had acHLA detected. There was no statistical significance in terms of sex, age, RBC count, platelet transfusion units, and drug treatments. 26/67 patients were homozygous HLA-A and -B and in this group, there is a statistical significance (p=0.007) when compared to heterozygotes. For 23 patients, they did EBM analysis on HLAMatchmaker, showing reactivity with public and private epitopes, with more antibodies against public epitopes. The most reactive eplets were 145QRT, 65QIA, 62QE, 127K, and 163EW. Only a single private epitope was found most frequently: 151AHA (A11). 15/23 had intra-CREG antibodies detected on public epitopes, 12/23 had ac against HLA-C detected. Analysis by epitope showed accuracy. All HLA-compatible platelets showed higher success than apheresis platelets, but there was no significance in the 24h ICCs between transfusions with A/B or CREG, or EBM compatible. To evaluate these different methods, 9 patients were chosen. Among these various methodologies, EBM was similar with A/B, and both were better than the CREG method. The order of methodology for compatibility in all immunized patients was defined as A/BU, followed by EBM and CREG. |
| 2013  EUA, CA | Transfusion Medicine | Low-level HLA antibodies do not predict platelet transfusion failure  in TRAP study participants | Jackman *et al*. | From the TRAP study, 170 ACL- subjects (70 clinically refractory, CR+ and 100 non-refractory, CR-) and 20 ACL+ subjects (10 CR+ and 10 CR-). 100/190 received leucoreduced platelet, 47/190 UV-treated platelet and 43/190 untreated PLT.  N.D. | Cross-sectional Retrospective | Luminex /LabScreen assay (One Lambda, Canoga Park, CA). Results with normalized background (normalized background, NBG). NGB > 10,8 (Class I HLA antibody) and NGB > 6,9 (HLA Class II antibody) were the cutoffs. | This work was supported by grants from the National Institutes of Health (grants R01HL-095470, R01HL-083388, and U01HL-42799). This manuscript was prepared using TRAP Research Materials obtained from the National Heart, Blood, and Lung  Institute Biologic Specimen and Data Repository Information Coordinating Center. | LCA+ patients had higher acHLA-I-II results than LCA- patients, some of whom had acHLA-I-II present with the new assay. With the adoption of statistical analysis by the ROC curve, the normalized background ratios for HLA-I predicted the LCA+ samples very well, becoming more moderate for HLA-II. Most participants in TRAP who developed PR were negative for acHLA when measured by LCA, which may have been because the assay was of low sensitivity. The study also presented data on acHLA persistence post-transfusion and a Kaplan-Meier survival statistical analysis was used, showing no statistical significance in acHLA-I-II persistence based on the patient's clinical refractory status. It is possible that some LCA- refractory individuals may have undetected antigens as rare antigens, or due to non-immunological refractory causes such as splenomegaly, sepsis, CIVD, venous-occlusive disease, and others. |
| 2013  Egypt | Transfusion and Apheresis Science | HLA alloimmunization inEgyptian aplastic anemia patients  receiving exclusively leukoreduced blood components | Enein *et al*. | 20 individuals with AA (14 men and 6 women) with congenital or acquired AA (10 congenital, 10 acquired)  N.D. | Cross-sectional Retrospective | FlowPRA screening kit, (OneLambda Canoga Park,USA). And complement-dependent cytotoxicity (CDC) assay. | No funding support inthe form ofgrants,equipme nts  or drugs was received | In this clinic, for over three years there has been a protocol of using single donor irradiated, CH leukoreduced platelets pre-storage. However, 5/20 received exclusively leukoreduced products due to disease (group 1). Platelets from a single donor were ABO incompatible in 94% of transfusions and were donated by family members. 6/20 were FlowPRA+, all from group 2. 4/6 were FlowPRA+ HLA-ClassI-II and 2/6 FlowPRA+ ClassII. All were male. All of group 1 were FlowPRA- and receiving leukoreduced CP may have contributed to this. HLA alloimmunization was not influenced by age. HLA-I-II alloimmunization was not influenced by the duration of transfusion, HLA-I was not influenced by the number of units of platelet concentrates and HEM transfused, but HLA-II had high numbers of CP and CH transfused (p<0.05). Fanconi patients showed a high incidence of acHLA (50%) compared to acquired (10%), but it was not statistically significant. 4/6 patients with FlowPRA+, had CDC+. 5 were false-positive by CDC. One patient died from intracranial hemorrhage with FlowPRA-I 65.7% and CDC-. 13/20 were refractory to platelets, but only 3/20 were FlowPRA-I+. There was refractoriness in group 1 (4/5) and group 2 (9/15). There was no correlation between FlowPRA-I-+ and PR (p<0.05). In evaluating the CDC technique, sensitivity and specificity were lacking. Disadvantages include the detection of not only acHLA but autoantibodies and non-specific antibodies and CDC PRAs may alter due to changes in the cellular composition of the panel or poor cell viability. FCM is more sensitive than CDC and can detect low titer antibodies as well as distinguish between IgG and IgM antibodies isotypes. |
| 2015  India | Asian Journal of Transfusion Science | Prevalence of risk factors for platelet transfusion  refractoriness in multitransfused hemato-oncological patients  at tertiary care center in North India | Kumawat *et al*. | 30 subjects (15 men and 15 women), 12 (7 men and 5 women) with AA, and 18 (8 men and 10 women) with AML (excluding LM3)  3 months of unspecified follow-up | Cross-sectional Retrospective | ELISA kit (Pakplus, GT diagnostic, USA) on three occasions (upon acceptance into the study, after 3 weeks or four transfusions, whichever occurred earlier, and at the end of 3 months) | N.D. | All patients had received transfusion prior to study participation (1-60 units, M=11). Most units were from random platelet donors. The refractory group required more CH and CP in all three phases of the study than the non-refractory group and was statistically significant. The association of acHLA and acHPA (HPA-5b) with transfusion PR was also significant (P≤ 0.05 and P=0.033, respectively), as was the non-immunological factor such as bleeding (P=0.019). Fever (P=0.08) and infection/sepsis (P=0.07) had no association. The presence of immune and non-immune factors were determinants in low platelet increment at the beginning of the study (P=0.021) and during the study (P ≤ 0.000), 4/13 non-refractory patients had acHLA while 14/17 refractory patients had the acHLA during the study (P ≤ 0.05). In this study, only one patient had splenomegaly, however, no correlations could be established. |
| 2015  Cuba | Correo Científico Médico de Holguín | Refractariedad a las transfusiones de plaquetas en pacientes con enfermedades oncológicas | Hernández *et al*. | 71 patients with oncological diseases (hematological, lung, breast, digestive tract, gynecological, soft tissue, and urinary tract)  Jul/2012 to Jul/2013 | Cross-sectional Prospective | Microlymphocytotoxicity for identification of HLA antibodies and Polyethylene glycol 6000 method for identification of circulating immune-complexes | N.D. | 14/71 patients were platelet refractory. Despite the predominance of females, gender was not statistically significant to be related to PR (p=0.31), besides age (p=0.1), although the highest percentage occurred between 35 and 59 years (not corroborating with the existence of pregnancy and senescence, respectively, described by the literature). The neoplastic origin was also unrelated (p=0.4), although, among refractory patients, the hematological origin was more frequent. And among the non-refractory patients, lung, breast, gynecologic, and hematologic neoplasms, respectively, also showed no significance. This did not agree with the literature that patients with onco hematologic neoplasms were transfused more frequently than those with other neoplasms. However, significance occurred when a transfusion history of CP was present in most refractory patients (p=0.01), in addition to fever (p=0.03), and not in the presence of splenomegaly and hemorrhage. Chemotherapy treatment also did not show statistical significance, but those with acHLA present did (p=0.01). The absence of significance was in agreement with the literature that mentions that radio/chemotherapy can suppress the bone marrow, decreasing the risk of platelet refractoriness. Previous transfusions of CP, from several different donors, corroborated the low platelet increment. In addition, high levels of circulating immunocomplexes were found in both groups and no anti-erythrocytic antibodies were detected. The absence of erythrocyte antibodies may have been due to ABO compatibility and the use of platelet concentrates less than 72 hours old. |
| 2016  EUA, WA | Vox Sanguinis | Clinical and laboratory correlates of  platelet alloimmunization and refractoriness in the  PLADO trial | Hess *et al*. | 816 included subjects (515 men, 106 multiparous women, and 195 women with ≥ 1 pregnancy), pediatric and adult subjects with hemato-oncological diseases  2004 to 2007 | Randomic | CCI and PRA HLA Class I (FlowPRA Screening Kit, One Lambda Corp,  Canoga Park, CA, USA); | Heart, Lung, and Blood Institute of the National Institutes of Health to the Data Coordinating Center at New England Research Institutes (HL072268), Case Western Reserve University (HL072033), Children’s Hospital Boston (HL072291), Cornell University (HL072196), Duke University (HL072289), Emory University (HL072248), Johns Hopkins University (HL072191), Puget Sound Blood Center (HL072305), Tulane university (HL072274), University of Iowa (HL072028), University of Maryland (HL072359), University of Minnesota (HL072072), University of North Carolina (HL072355), University of Oklahoma (HL072283), University of Pennsylvania (HL072346), University of Pittsburgh (HL072331) and the Blood Center of Wisconsin (HL072290). | PRA results ranging from -18 to 100% (m=2.7%) occurred. 45% were diagnosed with acute leukemia, 21% with lymphoma, and 12% with myeloma. There was no statistical significance between those who received different types of blood products (apheresis platelet concentrate - APBC - or buffy coat leukoreduced platelet concentrate - CPBCL) and the number of transfusions (P<0.001). 40/816 patients became alloimmunized. The type of blood component, the dose administered, and ABO compatibility were not significant with the incidence of alloimmunization (P<0.10), but low-dose platelets were (P<0.04). There was also no between treatment dose and type of blood component (P=0.95). Age did not influence (as it did for ICC), but women who had children were more alloimmunized than multiparous women and men (P<0.001). It was related to 9% of patients treated with chemotherapy, followed by 4% of those who underwent allogeneic bone marrow transplantation and 3% autologous or syngeneic. That is, it was more related in women who had already had children, in those who received low doses of platelets, and in those who underwent chemotherapy without bone marrow transplantation. A statistical relationship occurred in those who had transfusions of CPaf (15%) and CPBCL (23%) with ICC<5000, but not in successive counts from the same patient or at the first transfusion (P=0.73). This may be related to a group of patients who received many CPBCL transfusions showing frequent low PLT increment counts. The dose administered and ABO compatibility were significant in the occurrence of ICC<5000, both for the first transfusions and for the remaining ones. The type of hemocomponent did not influence until the emergence of low ICC or PR, but the number of transfusions and transplantation were associated with PR. |
| 2017  France | Leukemia Research | Platelet transfusion refractoriness in patients with acute myeloid leukemia  treated by intensive chemotherapy | Comont *et al*. | 41/897 cases of RP among AML patients who received intensive chemotherapy (excluding LM5)  Jan/2001 to Dec/2014 | Cross-sectional Prospective | Luminex/ LABScreen Mixed and Single Antigen of HLA Class I (One Lambda). Confirmed the most reactive with LCA-CDC using a 60-cell panel. | This study was supported  by grants from the French government under the  “Investissement d'avenir” program (ANR-11-PHUC-001). | Platelet and WBC count, respectively, at AML diagnosis, were low in subjects with PR (P=0.0002 and P=0.08). After the diagnosis of RP, 35/41 patients received CP from whole blood and all received CPaf. Molecular and cytogenetic abnormalities did not differ from AML patients without RP.  On multivariate analysis, the following were significantly statistically associated with RP: female gender (P=0.002), extramedullary involvement (P=0.002), and hemophagocytic syndrome during the induction phase (P=0.03). 18/31 patients with acHLA present had donor HLA compatibility and 15/18 received at least one transfusion of HLA-compatible CP during the induction phase. These concentrates induced a significant PLT increment in 37% of cases. Treatments were given for the management of RP including corticosteroids, intravenous immunoglobulins, TPO-RA, romiplostim and eltrombopag, rituximab, and bortezomib. TPO-RA did not contribute to lower platelet refractoriness or reduce grade-3-4 bleeding events or mortality. Patients with RP had more grade 3-4 bleeding events than those without RP during the chemotherapy induction phase (P<0.0001), more mortality (P<0.0001), and more incidence of bleeding deaths (P<0.0001). There was no survival rate relationship between the two groups (P=0.35). The groups with AML most susceptible to developing RP were as follows: women who have already had children, patients with extramedullary diseases, with low WBC counts, with infections at diagnosis, or with hemophagocytic syndrome. The therapeutic management of RP remains a challenge. Daily prophylactic transfusions are not recommended in cases of RP with immunological causes. Immunomodulators (corticosteroids, intravenous immunoglobulins, rituximab, or TPO-RA) need to be studied in a controlled study. But in this study, TPO-RA did not reduce the duration of platelet refractoriness or its complications, therefore, it is not recommended to use it in chemotherapy induction for AML patients. In addition, it is exposed that the mechanism of platelet clearance is not yet fully elucidated, although they cite treatment with abatacept in a murine model to treat RP due to HLA alloimmunization. |
| 2019  Boston, EUA | Am J Clin Pathol | Routine Solid Phase Multiplex Anti-HLA Antibody Tests  Predict Platelet Refractoriness | Peña *et al*. | 27 individuals (12 males and 15 females) with a diagnosis of ALL (1), AML/LMPA (17), SMD/DPM (3), MM (2), and LNH (4). 4/7 AlloPR presented with RFNH and no non-AlloPR. 6/20 non-AlloPR had an allergic reaction and only one AlloPR also  Jul/2013 to Dec/2015 | Cross-sectional Prospective | Screening performed with phenotyped beads from LABScreen PRA  (One Lambda Thermo Fisher; Luminex). If positive, single beads (LABScreen single antigen; One Lambda Thermo Fisher) were used. The strength of reactivity was reported as mean fluorescence intensity (MFI). PRA was calculated using Fusion software (One Lambda Thermo Fisher). Overall, MFI≥1000 was considered positive, although standard reactivity was also considered. The percent reactive antibody panel (ARPC) calculation was determined by the online calculator optn.transplant.hrsa.gov/converge/resources/allocationcalculators.asp | N.D. | 27 patients were tested before developing immune-caused RP (alloPR). After becoming thrombocytopenic, due to underlying disease or therapy, 10/27 developed RP, with 7/10 having a high level of HLA alloimmunization. 6/7 were women, all with documented pregnancies.  17 never developed RP and 3 became refractory with no appearance, according to the cut-off, of acHLA. 11/27 had a history of transfusion reactions that occurred at the end or after complete transfusion, except for one unit. In non-hemolytic febrile transfusion reactions, the ICC was <7.500. No impact of ABO incompatibility occurred in platelet transfusions, suggesting a little impact of isohemagglutinins, in this particular study.  Initial PRA was high in those who developed late alloPR compared to those who did not (P=0.002). 5/7 patients could have been predicted by using a cut-off of 30%. The study suggests that a high mean MFI of the 10 beads with the highest reactivities may predict the risk of developing alloPR.  As expected, the ICC of alloPR was significantly lower than non-alloPR (P<0.001) and the ICC of alloPR increased with HLA-compatible PLT compared to routine PLT (P=0.017). The initial PRA among those who developed RP was 44.6% (range = 0%-98%) and the time between first testing and RP was 24 days (range=6-241) and they received a mean of 6 (range=3-12) routine platelets. CPRA and/or mean MFI identified a cohort of patients at risk of developing a memory response to agHLA and hence PR. Patients in remission, under consolidation with high-dose cytarabine, with induction failure with refractory AML, and one under palliative chemotherapy with decitabine needed to be retested and CPRA calculations to be redone. The case of one patient who could not predict the development of alloPR illustrated that HLA alloimmunization evolves over time in response to allosensitization and that the diagnosis needs to be reconsidered even if initial tests are negative. |

Source: Elaborated by the authors (2022).
