# Supplementary material for: Anti-HLA antibody formation increases the chances of platelet refractoriness in platelet-transfused patients: a systematic review with meta-analysis
Source: Hematol Transfus Cell Ther. 2025 Apr 16;47(2):103821. doi: 10.1016/j.htct.2025.103821 (PMC12032179; doi:10.1016/j.htct.2025.103821)
Supplement: Supplementary file 2 [file mmc2.docx]

**Supplementary Table 3.** Risk of bias assessed by the Joanna Briggs Institute (JBI) Critical Appraisal Tools for use in JBI Critical Appraisal Checklist for Analytical Cross-Sectional Studies (JBI, 2020).

| Authors | Q1 | Q2 | Q3 | Q4 | Q5 | Q6 | Q7 | Q8 | %Yes | Risk |
| --- | --- | --- | --- | --- | --- | --- | --- | --- | --- | --- |
| Wu *et al*., 1976 | √ | -- | √ | √ | √ | √ | √ | √ | 87.5 | LRoB |
| Murphy *et al*., 1987 | √ | -- | √ | √ | √ | √ | √ | √ | 87.5 | LRoB |
| Godeau *et al*., 1992 | √ | -- | √ | √ | √ | √ | √ | √ | 87.5 | LRoB |
| Novotny *et al*., 1995 | √ | √ | √ | √ | √ | √ | √ | √ | 100 | LRoB |
| Bajpai *et al*., 2005 | √ | -- | √ | √ | √ | √ | √ | √ | 87.5 | LRoB |
| Lin *et al*., 2007 | √ | -- | √ | √ | √ | √ | √ | √ | 87.5 | LRoB |
| Pai *et al*., 2010 | √ | √ | √ | √ | √ | √ | √ | √ | 100 | LRoB |
| Jackman *et al*., 2013 | √ | -- | √ | √ | √ | √ | √ | √ | 87.5 | LRoB |
| Enein *et al*., 2013 | √ | -- | √ | √ | √ | √ | √ | √ | 87.5 | LRoB |
| Kumawat *et al*., 2015 | √ | -- | √ | √ | √ | √ | √ | √ | 87.5 | LRoB |
| Ramírez *et al*., 2015 | √ | √ | √ | √ | √ | √ | √ | √ | 100 | LRoB |
| Comont *et al*., 2017 | √ | √ | √ | √ | √ | √ | √ | √ | 100 | LRoB |
| Peña *et al*., 2019 | √ | √ | √ | √ | √ | √ | √ | √ | 100 | LRoB |

Source: Elaborated by the authors (2022).

Note: Q1. Were the criteria for inclusion in the sample clearly defined? Q2. Were the study subjects and the setting described in detail? Q3. Was the exposure measured validly and reliably? Q4. Were objective, standard criteria used for measurement of the condition? Q5. Were confounding factors identified? Q6. Were strategies to deal with confounding factors stated? Q7. Were the outcomes measured validly and reliably? Q8. Was appropriate statistical analysis used? √ - Yes; -- - No; U – Unclear; N/A – Not/Applicable. High risk of bias (HroB): ≤49%; moderate risk of bias (MroB): between 50% and 69%; low risk of bias(LroB): greater than 70% (Franco et al., 2020).

**Supplementary Table 4.** Risk of bias assessed by the Joanna Briggs Institute (JBI) Critical Appraisal Tools for use in JBI Critical Appraisal Checklist for Randomized Controlled Trials (JBI, 2020).

| Authors | Q1 | Q2 | Q3 | Q4 | Q5 | Q6 | Q7 | Q8 | Q9 | Q10 | Q11 | Q12 | Q13 | %Yes | Risk |
| --- | --- | --- | --- | --- | --- | --- | --- | --- | --- | --- | --- | --- | --- | --- | --- |
| Hess *et al*., 2016 | √ | √ | √ | U | U | U | U | √ | √ | √ | √ | √ | U | 61.5 | MroB |

Source: Elaborated by the authors (2022).

Note: Q1. Was true randomization used for assignment of participants to treatment groups? Q2. Was allocation to treatment groups concealed? Q3. Were treatment groups similar at the baseline? Q4. Were participants blind to treatment assignment? Q5. Were those delivering treatment blind to treatment assignment? Q6. Were outcomes assessors blind to treatment assignment? Q7. Were treatment groups treated identically other than the intervention of interest? Q8. Was follow up complete and if not, were differences between groups in terms of their follow up adequately described and analyzed? Q9. Were participants analyzed in the groups to which they were randomized? Q10. Were outcomes measured in the same way for treatment groups? Q11. Were outcomes measured reliably ? Q12. Was appropriate statistical analysis used? Q13. Was the trial design appropriate, and any deviations from the standard RCT design (individual randomization, parallel groups) accounted for in the conduct and analysis of the trial? √ - Yes; -- - No; U – Unclear; N/A - Not/Applicable. High risk of bias: ≤49%; moderate risk of bias: between 50% and 69%; low risk of bias: greater than 70% (Franco et al., 2020).

**Supplementary Table 5** – This table shows the publications without reported numbers of refractory individuals, and that it was not possible to perform a meta-analysis.

| Authors, Year | Technique | Refractoriness* | | Anti-HLA Antibodies | | Total (N) |
| --- | --- | --- | --- | --- | --- | --- |
|  |  | Present | Absent | Present | Absent |  |
| Killick *et al*., 1997 | LCT, ELISA (LCT negative) e LIFT | / | 24 | 11 | 13 | 24 |
| Kiefel *et al*., 2001 | LCT (PRA+ >20%) (Terasaki et al., 1964) and MAIPA | - | - | 108 | 144 | 252 |
| Imoto *et al*., 2007 | ELISA-Antigen Tray^TM^-LAT (One Lambda) from Oct/2000 to Sep/2004 and LAB-Screen PRA^R^/Luminex 100^TM^ IS System from Oct/2004 to July/2005 | - | - | 50 | 173 | 223 |
| Arruda *et al*., 2008 | µ-LCT and PRA (Scornick et al, 1992) followed by ELISA-LAT^M^ (to confirm HLA-Class I and II) | - | - | 38 | 72 | 110 |
| Imoto *et al*., 2010 | ELISA (Lambda Antigen Tray™ (LAT); One Lambda, Inc., Canoga Park, CA, USA) / LAT-1240 and method Luminex using LABScreen® Single Antigen (One Lambda)/ Luminex 100 IS System (Luminex Corporation, Austin, TX, USA) | - | - | 35 | 72 | 107 |
| Wang; Xia *et al*., 2017 | Anti-HLA antibody screening technique: ELISA by Lifecodes PAKPLUS (Immucor GTI Diagnostics, Inc., Waukesha, WI, USA). Detecting specificities: Luminex Single Antigen Class I (LSAClass I, GEN-PROBE, Lifecodes, USA). Analyzing crossmatching: MASPAT kit (Monoclonal Antibody Solid-phase Platelet Antibody Test, Sanquin, Amsterdam, the Netherlands). | 204 | / | 110 | 94 | 204 |
| Abraham *et al*., 2018 | PAK- 2LE kiT (Immucor GTi E) and Luminex bead-based assay (Lifecodes Life screen Deluxe – LMX) | 80 | / | 15 | 65 | 80 |
| Kallon *et al*., 2019 | PCR/SSO. Luminex® (One Lambda). CDC in house. MFI cutoff = 1500 only for new patients (the old ones had only CDC results). Triplet mismatches were investigated by HLAMatchmaker (VersionSER1.3.xls) at w[ww.epitopes.](http://ww.epitopes.)net, while Eplet mismatches were detected by HLAMatchmaker (ABC Eplet Matching Macro v2.1_)q.xls). The use of medium-resolution to high-resolution conversion (96.43% accuracy for HLA-Class I) was adopted. | 37 | / | 37 | 0 | 37 |
| Fagundes *et al*., 2020 | LABScreen®, Single Antigen, (One Lambda Inc.). Fusion 4.3 (One Lambda, Inc) with MFI ≥ 1000 (cutoff point). SSO method (Labtype-A, B, C, One Lambda Inc.) from donors. Software developed on Access platform (Microsoft, Inc) to register and select 500 unrelated platelet donors. | 75 | / | 50 | 25 | 75 |
| Coombs; Hassen *et al*., 2020 | LABScreen Single Antigen Class I (One Lambda, Thermo Fisher Scientific, Waltham, MA). Results were positive if MFI was higher than negative controls (plasma from healthy donors). Also noted when MFI of 1500, which is required by FCA in a grafting protocol. Flow Cytometry (LSRFortessa, BD Biosciences, Franklin Lakes, NJ) analyzed with FlowJo version 10.5.3 software (BD Biosciences) | - | - | 124 | 0 | 124 |
| Juskewitch *et al*., 2021 | HLA typing (LABType SSO, One Lambda, West Hills, CA). Platelet Cross Testing (Capture-P,  Immucor, Norcross, GA), using EDTA-treated patient plasma against 6-14 units of ABO-compatible CPaf with reduced pathogens (Intercept, Cerus, Concord, CA) or irradiated. Detection of Class I anti-HLA antibodies by LABScreen Single Antigen (One Lambda, West Hills, CA). Because antibodies can decline, Class I HLA and HLA antibody results were only considered within 30 days of cross-testing.. | 59 | / | 4 | 55 | 59 |
| Ma *et al*., 2022 | LABScreen Mixed Kit (One Lambda, Canoga Park, CA). LABScreen Single Antigen beads assay (One Lambda). MFI was calculated from normalization with the negative control. The HLA-A, -B, -C, -DR, -DQ, and -DP loci were evaluated. | - | - | 1040 | 2765 | 3805 |

Source: Elaborated by the authors (2022).

Note: *Fields with the symbol (-) were the studies in which the authors did not report the number of refractory patients. The fields filled with the symbol (/) were those of which it was not possible to perform the meta-analysis.
